# 1-(10*H*-Phenothia­zin-2-yl)ethanone

**DOI:** 10.1107/S1600536811001851

**Published:** 2011-01-22

**Authors:** Jerry P. Jasinski, Albert E. Pek, Prakash S. Nayak, B. Narayana, H. S. Yathirajan

**Affiliations:** a Department of Chemistry, Keene State College, 229 Main Street, Keene, NH 03435-2001, USA; b Department of Studies in Chemistry, Mangalore University, Mangalagangotri 574 199, India; c Department of Studies in Chemistry, University of Mysore, Manasagangotri, Mysore 570 006, India

## Abstract

In the title compound, C_14_H_11_NOS, the thia­zine ring adopts a slightly distorted boat conformation. The dihedral angle between the mean planes of the two benzene rings is 20.2 (9)°. An inter­molecular N—H⋯O hydrogen bond and a weak C—H⋯π inter­action occur in the crystal, creating a two-dimensional network parallel to the *bc* plane.

## Related literature

For applications of phenothia­zines in drugs and medicine, see: Miller *et al.* (1999 ▶); Wermuth (2003 ▶); Wang *et al.* (2008 ▶); Lam *et al.* (2001 ▶); Kojilo *et al.* (2001 ▶). For related structures, see: Bell *et al.* (1968 ▶); McDowell (1969 ▶, 1970 ▶, 1975 ▶, 1976 ▶, 1978 ▶, 1980 ▶); Chu & Van der Helm (1974 ▶, 1975 ▶, 1977) ▶); Phelps & Cordes (1974 ▶, 1975 ▶); Harrison *et al.* (2007 ▶); Wang *et al.* (2009 ▶). For puckering parameters, see: Cremer & Pople (1975 ▶).
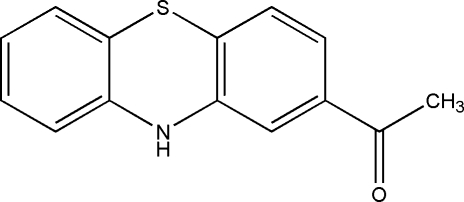


## Experimental

### 

#### Crystal data

C_14_H_11_NOS
*M* *_r_* = 241.30
Monoclinic, 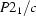

*a* = 14.3445 (18) Å
*b* = 5.5425 (7) Å
*c* = 15.694 (2) Å
β = 114.494 (2)°
*V* = 1135.4 (2) Å^3^
*Z* = 4
Mo *K*α radiation
μ = 0.27 mm^−1^
*T* = 100 K
0.55 × 0.55 × 0.10 mm

#### Data collection

Bruker APEXII CCD diffractometer
Absorption correction: multi-scan (*SADABS*; Bruker, 2008 ▶) *T*_min_ = 0.868, *T*_max_ = 0.974
8194 measured reflections
3331 independent reflections
2828 reflections with *I* > 2σ(*I*)
*R*_int_ = 0.025

#### Refinement

*R*[*F* ^2^ > 2σ(*F* ^2^)] = 0.038
*wR*(*F* ^2^) = 0.108
*S* = 1.04
3331 reflections
159 parameters
H atoms treated by a mixture of independent and constrained refinement
Δρ_max_ = 0.49 e Å^−3^
Δρ_min_ = −0.29 e Å^−3^

### 

Data collection: *APEX2* (Bruker, 2008 ▶); cell refinement: *SAINT* (Bruker, 2008 ▶); data reduction: *SAINT*; program(s) used to solve structure: *SHELXS97* (Sheldrick, 2008 ▶); program(s) used to refine structure: *SHELXTL* (Sheldrick, 2008 ▶); molecular graphics: *SHELXTL*; software used to prepare material for publication: *SHELXTL*.

## Supplementary Material

Crystal structure: contains datablocks global, I. DOI: 10.1107/S1600536811001851/is2662sup1.cif

Structure factors: contains datablocks I. DOI: 10.1107/S1600536811001851/is2662Isup2.hkl

Additional supplementary materials:  crystallographic information; 3D view; checkCIF report

## Figures and Tables

**Table 1:** Hydrogen-bond geometry (Å, °) *Cg*3 is the centroid of the C7–C12 ring.

*D*—H⋯*A*	*D*—H	H⋯*A*	*D*⋯*A*	*D*—H⋯*A*
N1—H15⋯O1^i^	0.829 (18)	2.198 (18)	3.0042 (15)	164.3 (17)
C9—H14⋯*Cg*3^ii^	0.93	2.64	3.306 (7)	130

Symmetry codes: (i) 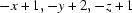; (ii) 
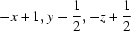
.

**Fig. 1.**
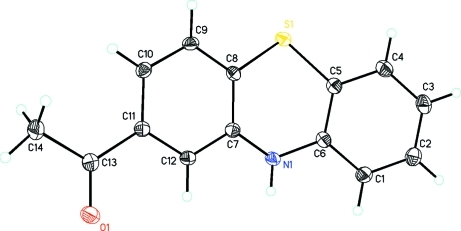
Molecular structure of C14H11N O S, showing the atom labeling scheme and 50% probability displacement ellipsoids.

**Fig. 2.**
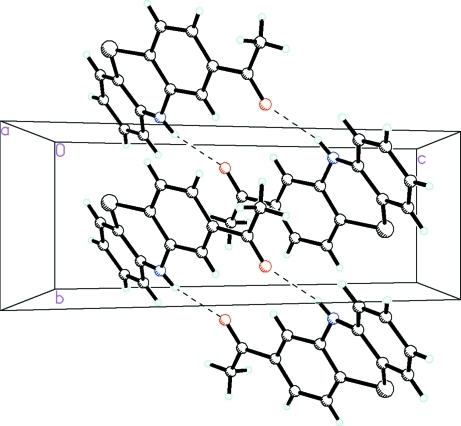
Packing diagram of the title compound viewed down the a axis. Dashed lines indicate N—H···O hydrogen bonding creating a 2-D network structure parallel to the bc plane.

## supplementary crystallographic information

### Comment

Phenothiazine is a well known heterocycle. The phenothiazine structure occurs in many synthetic dyes, electroluminescent materials (Miller *et al.*, 1999) and drugs, especially various antipsychotic drugs, *e.g.* chlorpromazine and antihistaminic drugs, *e.g.* promethazine (Wermuth, 2003). Recently, researchers have found new applications for phenothiazine derivatives in medicine related to antitubercular (Wang *et al.*, 2008) and antitumor activities (Lam *et al.*, 2001). A review of various aspects of phenothiazines has been published (Kojilo *et al.*, 2001). The crystal and molecular structure studies of phenothiazine (Bell *et al.*, 1968), chlorpromazine, thiethylperazine, thioridazine, phenothiazine, perphenazine, trifluperazine hydrochloride (McDowell, 1969, 1970, 1975, 1976, 1978, 1980), *N*-methylphenothiazine, *N*-ethylphenothiazine, *N*-benzylphenothiazine (Chu & Van der Helm, 1974, 1975, 1977), triflupromazine, 2-methoxyphenothiazine (Phelps & Cordes, 1974, 1975), Phenothiazine-picric acid (1/1) (Harrison *et al.*, 2007) and 10-acetyl-10*H*-phenothiazine 5-oxide (Wang *et al.*, 2009) have been reported. In view of the importance of phenothiazines, this paper reports the crystal structure of the title compound, 1-(10*H*-phenothiazin-2-yl)ethanone.

The title compound, C_14_H_11_NOS, consists of benzene and phenyl-ethanone rings fused to a thiazine ring which adopts a slightly distorted boat conformation with puckering parameters Q, θ and φ of 0.371 (4) Å, 100.1 (2)° and 181.457 (4)°, respectively (Cremer & Pople, 1975) (Fig. 1). For an ideal boat Phi= k *x* 60. The dihedral angles between the mean planes of the two 6-membered benzene rings, and thiazine ring are 10.5 (5) and 10.3 (6)°. An N—H···O intermolecular hydrogen bond and a weak C—H···π interaction (Table 1) contributes to crystal packing creating a 2-D network structure parallel to the *bc* plane (Fig. 2).

### Experimental

1-(10*H*-Phenothiazin-3-yl)ethanone was obtained from Aldrich and it was crystallized from a dimethylformamide solution (m.p. 455–457 K)

### Refinement

The H15 atom bonded to N1 was freely refined. All of the other H atoms were placed in their calculated positions and then refined using the riding model with C—H lengths of 0.93 Å (CH), or 0.96 Å (CH_3_). Isotropic displacement parameters for these atoms were set to 1.18–1.20 (CH) or 1.51 (CH_3_) times *U*_eq_ of the parent atom.

### Crystal data

**Table:**

C_14_H_11_NOS	*F*(000) = 504
*M**_r_* = 241.30	*D*_x_ = 1.412 Mg m^−^^3^
Monoclinic, *P*2_1_/*c*	Mo *K*α radiation, λ = 0.71073 Å
Hall symbol: -P 2ybc	Cell parameters from 2781 reflections
*a* = 14.3445 (18) Å	θ = 2.6–30.6°
*b* = 5.5425 (7) Å	µ = 0.27 mm^−^^1^
*c* = 15.694 (2) Å	*T* = 100 K
β = 114.494 (2)°	Plate, orange
*V* = 1135.4 (2) Å^3^	0.55 × 0.55 × 0.10 mm
*Z* = 4	

### Data collection

**Table:**

Bruker APEXII CCD diffractometer	3331 independent reflections
Radiation source: fine-focus sealed tube	2828 reflections with *I* > 2σ(*I*)
graphite	*R*_int_ = 0.025
ω scans	θ_max_ = 31.2°, θ_min_ = 1.6°
Absorption correction: multi-scan (*SADABS*; Bruker, 2008)	*h* = −20→18
*T*_min_ = 0.868, *T*_max_ = 0.974	*k* = −8→7
8194 measured reflections	*l* = −21→22

### Refinement

**Table:**

Refinement on *F*^2^	Primary atom site location: structure-invariant direct methods
Least-squares matrix: full	Secondary atom site location: difference Fourier map
*R*[*F*^2^ > 2σ(*F*^2^)] = 0.038	Hydrogen site location: inferred from neighbouring sites
*wR*(*F*^2^) = 0.108	H atoms treated by a mixture of independent and constrained refinement
*S* = 1.04	*w* = 1/[σ^2^(*F*_o_^2^) + (0.0553*P*)^2^ + 0.4353*P*] where *P* = (*F*_o_^2^ + 2*F*_c_^2^)/3
3331 reflections	(Δ/σ)_max_ < 0.001
159 parameters	Δρ_max_ = 0.49 e Å^−^^3^
0 restraints	Δρ_min_ = −0.29 e Å^−^^3^

### Special details

**Table:**

Geometry. All e.s.d.'s (except the e.s.d. in the dihedral angle between two l.s. planes) are estimated using the full covariance matrix. The cell e.s.d.'s are taken into account individually in the estimation of e.s.d.'s in distances, angles and torsion angles; correlations between e.s.d.'s in cell parameters are only used when they are defined by crystal symmetry. An approximate (isotropic) treatment of cell e.s.d.'s is used for estimating e.s.d.'s involving l.s. planes.
Refinement. Refinement of *F*^2^ against ALL reflections. The weighted *R*-factor *wR* and goodness of fit *S* are based on *F*^2^, conventional *R*-factors *R* are based on *F*, with *F* set to zero for negative *F*^2^. The threshold expression of *F*^2^ > σ(*F*^2^) is used only for calculating *R*-factors(gt) *etc*. and is not relevant to the choice of reflections for refinement. *R*-factors based on *F*^2^ are statistically about twice as large as those based on *F*, and *R*- factors based on ALL data will be even larger.

### Fractional atomic coordinates and isotropic or equivalent isotropic displacement parameters (Å^2^)

**Table:**

	*x*	*y*	*z*	*U*_iso_*/*U*_eq_	
S1	0.26560 (3)	0.41617 (6)	0.15897 (2)	0.01761 (10)	
O1	0.65447 (8)	0.81327 (19)	0.54988 (7)	0.0213 (2)	
N1	0.30331 (9)	0.8588 (2)	0.28579 (8)	0.0165 (2)	
C11	0.55267 (10)	0.5672 (2)	0.42245 (9)	0.0140 (2)	
C8	0.37282 (10)	0.4732 (2)	0.26461 (9)	0.0139 (2)	
C12	0.47152 (10)	0.7316 (2)	0.39393 (9)	0.0143 (2)	
H11	0.4778	0.8723	0.4282	0.017*	
C13	0.64777 (10)	0.6258 (2)	0.50679 (9)	0.0158 (3)	
C5	0.17254 (10)	0.5996 (2)	0.17352 (9)	0.0158 (3)	
C7	0.38142 (10)	0.6876 (2)	0.31481 (9)	0.0137 (2)	
C10	0.54264 (10)	0.3531 (2)	0.37268 (9)	0.0148 (2)	
H9	0.5960	0.2420	0.3918	0.018*	
C6	0.20076 (10)	0.8030 (2)	0.23161 (9)	0.0154 (2)	
C14	0.73624 (11)	0.4520 (3)	0.53624 (10)	0.0212 (3)	
H13A	0.7645	0.4522	0.4905	0.032*	
H13B	0.7127	0.2927	0.5412	0.032*	
H13C	0.7879	0.5003	0.5959	0.032*	
C2	0.02171 (11)	0.9054 (3)	0.17891 (11)	0.0228 (3)	
H2	−0.0286	1.0098	0.1799	0.027*	
C4	0.06974 (11)	0.5477 (3)	0.12089 (10)	0.0201 (3)	
H4	0.0514	0.4087	0.0845	0.024*	
C1	0.12425 (11)	0.9539 (3)	0.23424 (10)	0.0199 (3)	
H1	0.1420	1.0881	0.2733	0.024*	
C3	−0.00598 (11)	0.7028 (3)	0.12236 (10)	0.0231 (3)	
H3	−0.0747	0.6703	0.0856	0.028*	
C9	0.45264 (10)	0.3070 (2)	0.29454 (9)	0.0150 (2)	
H14	0.4456	0.1634	0.2618	0.018*	
H15	0.3129 (13)	0.971 (3)	0.3234 (12)	0.018 (4)*	

### Atomic displacement parameters (Å^2^)

**Table:**

	*U*^11^	*U*^22^	*U*^33^	*U*^12^	*U*^13^	*U*^23^
S1	0.01602 (18)	0.01739 (18)	0.01652 (17)	−0.00026 (12)	0.00384 (13)	−0.00450 (11)
O1	0.0212 (5)	0.0192 (5)	0.0192 (5)	−0.0004 (4)	0.0041 (4)	−0.0037 (4)
N1	0.0149 (5)	0.0108 (5)	0.0202 (5)	0.0003 (4)	0.0036 (4)	−0.0024 (4)
C11	0.0145 (6)	0.0128 (6)	0.0148 (5)	−0.0002 (4)	0.0061 (5)	0.0008 (4)
C8	0.0137 (6)	0.0131 (6)	0.0150 (5)	−0.0022 (4)	0.0059 (5)	−0.0003 (4)
C12	0.0162 (6)	0.0112 (6)	0.0156 (6)	−0.0004 (4)	0.0067 (5)	−0.0008 (4)
C13	0.0159 (6)	0.0159 (6)	0.0160 (6)	−0.0009 (5)	0.0070 (5)	0.0015 (5)
C5	0.0155 (6)	0.0153 (6)	0.0150 (6)	0.0007 (5)	0.0045 (5)	0.0019 (4)
C7	0.0147 (6)	0.0113 (6)	0.0155 (6)	−0.0003 (5)	0.0065 (5)	0.0012 (4)
C10	0.0150 (6)	0.0124 (6)	0.0177 (6)	0.0014 (5)	0.0074 (5)	0.0012 (5)
C6	0.0147 (6)	0.0142 (6)	0.0154 (6)	−0.0003 (5)	0.0042 (5)	0.0018 (4)
C14	0.0158 (7)	0.0205 (7)	0.0233 (7)	0.0024 (5)	0.0041 (6)	0.0005 (5)
C2	0.0164 (7)	0.0238 (7)	0.0252 (7)	0.0043 (5)	0.0055 (6)	−0.0004 (5)
C4	0.0189 (7)	0.0203 (7)	0.0177 (6)	−0.0022 (5)	0.0043 (5)	−0.0019 (5)
C1	0.0181 (7)	0.0179 (7)	0.0219 (6)	0.0019 (5)	0.0063 (6)	−0.0020 (5)
C3	0.0141 (6)	0.0273 (8)	0.0225 (7)	−0.0005 (5)	0.0022 (5)	−0.0011 (6)
C9	0.0175 (6)	0.0104 (6)	0.0190 (6)	−0.0007 (5)	0.0096 (5)	−0.0015 (4)

### Geometric parameters (Å, °)

**Table:**

S1—C8	1.7606 (13)	C5—C6	1.4003 (18)
S1—C5	1.7664 (14)	C10—C9	1.3868 (18)
O1—C13	1.2216 (16)	C10—H9	0.9300
N1—C7	1.3930 (16)	C6—C1	1.3939 (18)
N1—C6	1.3948 (17)	C14—H13A	0.9600
N1—H15	0.829 (18)	C14—H13B	0.9600
C11—C10	1.3946 (18)	C14—H13C	0.9600
C11—C12	1.3980 (18)	C2—C3	1.384 (2)
C11—C13	1.4903 (18)	C2—C1	1.390 (2)
C8—C9	1.3908 (18)	C2—H2	0.9300
C8—C7	1.4026 (18)	C4—C3	1.393 (2)
C12—C7	1.3932 (18)	C4—H4	0.9300
C12—H11	0.9300	C1—H1	0.9300
C13—C14	1.5051 (19)	C3—H3	0.9300
C5—C4	1.3891 (19)	C9—H14	0.9300
			
C8—S1—C5	100.77 (6)	C1—C6—N1	119.56 (12)
C7—N1—C6	123.39 (11)	C1—C6—C5	118.93 (12)
C7—N1—H15	113.9 (12)	N1—C6—C5	121.51 (12)
C6—N1—H15	115.0 (12)	C13—C14—H13A	109.5
C10—C11—C12	119.76 (12)	C13—C14—H13B	109.5
C10—C11—C13	121.79 (12)	H13A—C14—H13B	109.5
C12—C11—C13	118.44 (11)	C13—C14—H13C	109.5
C9—C8—C7	120.20 (12)	H13A—C14—H13C	109.5
C9—C8—S1	118.33 (10)	H13B—C14—H13C	109.5
C7—C8—S1	121.34 (10)	C3—C2—C1	120.39 (13)
C7—C12—C11	120.84 (12)	C3—C2—H2	119.8
C7—C12—H11	119.6	C1—C2—H2	119.8
C11—C12—H11	119.6	C5—C4—C3	120.36 (13)
O1—C13—C11	120.67 (12)	C5—C4—H4	119.8
O1—C13—C14	120.76 (12)	C3—C4—H4	119.8
C11—C13—C14	118.55 (12)	C2—C1—C6	120.52 (13)
C4—C5—C6	120.19 (12)	C2—C1—H1	119.7
C4—C5—S1	118.48 (10)	C6—C1—H1	119.7
C6—C5—S1	121.18 (10)	C2—C3—C4	119.54 (13)
N1—C7—C12	119.68 (11)	C2—C3—H3	120.2
N1—C7—C8	121.47 (12)	C4—C3—H3	120.2
C12—C7—C8	118.83 (12)	C10—C9—C8	120.71 (12)
C9—C10—C11	119.62 (12)	C10—C9—H14	119.6
C9—C10—H9	120.2	C8—C9—H14	119.6
C11—C10—H9	120.2		
			
C5—S1—C8—C9	159.37 (10)	C12—C11—C10—C9	0.86 (18)
C5—S1—C8—C7	−24.72 (12)	C13—C11—C10—C9	−179.90 (11)
C10—C11—C12—C7	−1.68 (18)	C7—N1—C6—C1	156.10 (12)
C13—C11—C12—C7	179.06 (11)	C7—N1—C6—C5	−24.60 (19)
C10—C11—C13—O1	−179.23 (12)	C4—C5—C6—C1	−1.17 (19)
C12—C11—C13—O1	0.02 (18)	S1—C5—C6—C1	174.31 (10)
C10—C11—C13—C14	2.26 (18)	C4—C5—C6—N1	179.53 (13)
C12—C11—C13—C14	−178.50 (12)	S1—C5—C6—N1	−5.00 (18)
C8—S1—C5—C4	−158.90 (11)	C6—C5—C4—C3	2.7 (2)
C8—S1—C5—C6	25.55 (12)	S1—C5—C4—C3	−172.88 (11)
C6—N1—C7—C12	−156.23 (12)	C3—C2—C1—C6	1.8 (2)
C6—N1—C7—C8	25.55 (19)	N1—C6—C1—C2	178.23 (13)
C11—C12—C7—N1	−177.47 (11)	C5—C6—C1—C2	−1.1 (2)
C11—C12—C7—C8	0.80 (18)	C1—C2—C3—C4	−0.3 (2)
C9—C8—C7—N1	179.12 (11)	C5—C4—C3—C2	−2.0 (2)
S1—C8—C7—N1	3.28 (17)	C11—C10—C9—C8	0.82 (19)
C9—C8—C7—C12	0.89 (18)	C7—C8—C9—C10	−1.71 (19)
S1—C8—C7—C12	−174.95 (9)	S1—C8—C9—C10	174.25 (10)

### Hydrogen-bond geometry (Å, °)

**Table:**

Cg3 is the centroid of the C7–C12 ring.

**Table:**

*D*—H···*A*	*D*—H	H···*A*	*D*···*A*	*D*—H···*A*
N1—H15···O1^i^	0.829 (18)	2.198 (18)	3.0042 (15)	164.3 (17)
C9—H14···Cg3^ii^	0.93	2.64	3.306 (7)	130

Symmetry codes: (i) −*x*+1, −*y*+2, −*z*+1; (ii) −*x*+1, *y*−1/2, −*z*+1/2.
